# Electronic structure and correlations in planar trilayer nickelate Pr_4_Ni_3_O_8_

**DOI:** 10.1126/sciadv.ade4418

**Published:** 2023-01-13

**Authors:** Haoxiang Li, Peipei Hao, Junjie Zhang, Kyle Gordon, A. Garrison Linn, Xinglong Chen, Hong Zheng, Xiaoqing Zhou, J. F. Mitchell, D. S. Dessau

**Affiliations:** ^1^ Department of Physics, University of Colorado Boulder, Boulder, CO 80309, USA.; ^2^ Advanced Materials Thrust, The Hong Kong University of Science and Technology (Guangzhou), Guangzhou, Guangdong 511453, China.; ^3^ Materials Science Division, Argonne National Laboratory, Lemont, IL 60439, USA.; ^4^ Institute of Crystal Materials and State Key Laboratory of Crystal Materials, Shandong University, Jinan, Shandong 250100, China.; ^5^ Center for Experiments on Quantum Materials, University of Colorado Boulder, Boulder, CO 80309, USA.

## Abstract

The discovery of superconductivity in planar nickelates raises the question of how the electronic structure and correlations of Ni^1+^ compounds compare to those of the Cu^2+^ cuprate superconductors. Here, we present an angle-resolved photoemission spectroscopy (ARPES) study of the trilayer nickelate Pr_4_Ni_3_O_8_, revealing a Fermi surface resembling that of the hole-doped cuprates but with critical differences. Specifically, the main portions of the Fermi surface are extremely similar to that of the bilayer cuprates, with an additional piece that can accommodate additional hole doping. We find that the electronic correlations are about twice as strong in the nickelates and are almost *k*-independent, indicating that they originate from a local effect, likely the Mott interaction, whereas cuprate interactions are somewhat less local. Nevertheless, the nickelates still demonstrate the strange-metal behavior in the electron scattering rates. Understanding the similarities and differences between these two families of strongly correlated superconductors is an important challenge.

## Teaser

Cuprate-like electronic structure and exotic many body interactions are found in planar nickelates.

## INTRODUCTION

As 3*d* transition metal oxides, various nickelate compounds have long been studied for correlated electron properties and as candidates for high-*T*_C_ superconductivity. Special attention has been paid to two-dimensional Ni^1+^ compounds that might mimic the Cu^2+^ state of the cuprates that is known to support both the strange-metal physics and high-*T*_C_ superconductivity. However, even with the same formal electron count between the Ni^1+^ and Cu^2+^ states, concerns have been raised that these two states would not be the same and so could not support the same exotic physics as the cuprates, with concerns arising due to reduced 3*d*-2*p* mixing and different on-site correlation energies (*1*, *2*). These differences could arise due to the varying energetics of the average transition metal *d* bands as a function of atomic number. Described in the Zaanen-Sawatzky-Allen classification schemes (*3*), correlation gaps arise from Mott correlations that principally center on the transition metal (farther left in the periodic table) or charge-transfer physics (farther right) that includes the oxygen ligands more explicitly and brings in the Zhang-Rice singlet that is a combination of the Cu and O orbitals (*4*).

Recently, the spotlight has been turned to square-planar nickelate materials following the breakthrough discovery of superconductivity in thin films of the planar infinite layer nickelates, Sr/Ca-doped RNiO_2_ (R = Pr, Nd, La) (*5*–*8*), and, more recently, the five-layer variant, Nd_6_Ni_5_O_12_ (*9*). Proposals have been put forward that the superconducting nickelate should express exotic electronic correlations similar to those found in other unconventional superconductors, such as the doped charge-transfer cuprates (*10*–*14*). However, the reduction process used in the synthesis of these nickelate thin films, which is required to remove the apical oxygen atoms and thus to create the square planar network, is known to degrade the surface. This has so far prohibited investigations necessary to make direct measurements of the electronic structure and test these conjectures because the most direct probes of the electronic structure and carrier dynamics [e.g., angle-resolved photoemission spectroscopy (ARPES)] require surface-sensitive techniques.

**Fig. 1.**
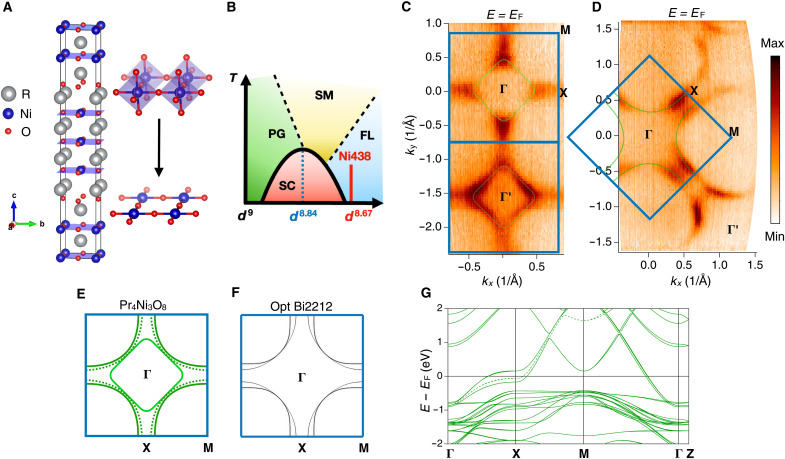
Crystal structure and Fermi surface of Pr_4_Ni_3_O_8_. (**A**) Unit cell of Pr_4_Ni_3_O_8_ and a schematic showing the change from layered perovskite structure to square planar structure by the reduction of the apical oxygen. (**B**) Simplified doping phase diagram of cuprate superconductors, with the planar trilayer nickelate doping level (red line) residing in the overdoped region. SC, superconductivity; PG, pseudogap; SM, strange metal; FL, Fermi liquid. (**C**) Fermi surface maps taken with 84-eV photons that emphasize the electron pocket around the Γ point. (**D**) Fermi surface map taken with 55-eV photons, which emphasize the hole pocket centered around the zone corners. (**E**) Schematics of the Fermi surface. The solid green pockets indicate the electron and hole pockets shown in (C) and (D). The dashed hole pocket is an extra band shown in DFT result [see (G)]. Two of the three Fermi surfaces are extremely similar to those of cuprates, including effective doping level. The third band in the nickelate accommodates extra holes. (**F**) A simulated Fermi surface of an optimally doped Bi_2_Sr_2_CaCu_2_O_8+*x*_ (Bi2212) with the tight binding model parameters from (*45*). (**G**) Calculated band structure using the GGA method. Here, we show the DFT result of La_4_Ni_3_O_8_ instead of Pr_4_Ni_3_O_8_ to avoid the issues related to the Pr-4*f* states. The DFT results of Pr_4_Ni_3_O_8_ are shown in fig. S5, and the issue with the Pr-4*f* is discussed in the Materials and Methods and Supplementary Text S7, and in another DFT paper on this material (*23*).

We focus on the planar trilayer nickelates Pr_4_Ni_3_O_8_ that can be grown in bulk, cleavable crystalline form suitable for ARPES (*15*). In this material, the apical oxygen has been removed from the Ruddlesden-Popper (R-P) phase Pr_4_Ni_3_O_10_ structure by reduction, leading to square planar nickel assembled into triple NiO_2_ layers that alternate with fluorite-like R_2_O_2_ layers (Fig. 1A). In contrast to the R-P nickelates such as RNiO_3_, R_2_NiO_4_, and R_4_Ni_3_O_10_ that several previous studies have focused on (*16*–*21*), the planar nickelates, e.g., R_1−*x*_Sr*_x_*NiO_2_ and R_4_Ni_3_O_8_, are proximate to the *d*^9^ electron count per transition metal ion that is common to the cuprates. On the basis of the electron counting of the 3*d* orbital states, the planar trilayer nickelates have a one-third–hole doping with average orbital occupancy *d*^8.67^, which nominally is on the heavily overdoped side of the high-*T*_C_ cuprate phase diagram (Fig. 1B). Furthermore, a recent study has revealed a large orbital polarization of *d_x_*_^2^__-*y*^2^_ character in the 3*d* conduction bands of R_4_Ni_3_O_8_––a key signature of the cuprate electronic structure (*22*). Nevertheless, no direct experimental measurement of the electronic structure and carrier dynamics of these materials has yet been reported.

## RESULTS

We performed high-resolution ARPES measurements on single crystals of trilayer nickelate Pr_4_Ni_3_O_8_, cleaved in situ in the ultrahigh vacuum environment of the ARPES spectrometers. To explore the electronic structure, we first present the Fermi surface measured at *T* = 22 K in Fig. 1 (C and D). By using different photon energies (84 and 55 eV), we take advantage of the matrix element effects to highlight distinct parts of the Fermi surface (see fig. S10 for additional data taken with different combinations of photon energies, polarizations, and experimental geometries). The Fermi surface maps display an electron pocket centered at Γ (first zone) and Γ′ (second zone) (Fig. 1C) and a hole pocket or pockets centered around the zone corner (Fig. 1D). Pr_4_Ni_3_O_8_ has a much simpler Fermi surface than that of the related trilayer (R-P) nickelate La_4_Ni_3_O_10_, which has a substantially different electron count, *d*^7.33^ (*16*), and an additional piece of Fermi surface near the Γ point not found in either the cuprates or the present compound. In Fig. 1E, we sketch the two main Fermi pockets from panels (C) and (D) in solid line while including a third pocket in dashed line that is more difficult to resolve from those experimental panels. We compare these results with the Fermi surface of optimally doped bilayer cuprate Bi_2_Sr_2_CaCu_2_O_8+*x*_ (Bi2212) (Fig. 1F). We see that the Fermi surface topologies are extremely similar, except for the additional inner piece of Fermi surface of Pr_4_Ni_3_O_8_ that accommodates the extra doping of holes in that material. The density functional theory (DFT) result further supports our findings from ARPES (Fig. 1G). However, to avoid issues related to the 4*f* states of Pr in the DFT calculation that contradicts the experiment results (see Materials and Methods and Supplementary Text S7) (*22*), we perform the DFT calculations on the closely related compound La_4_Ni_3_O_8_ instead, an approach taken in another DFT calculations on this material (*23*) and on the infinite layer nickelate (*24*). Here, we note that both La and Pr are expected to be in the 3+ valence state, and so, the valence of Ni and O in Pr_4_Ni_3_O_8_ and La_4_Ni_3_O_8_ are expected to be extremely similar—something that is confirmed by the excellent agreement between the experimental and theoretical Fermi surfaces shown here. It is worth noting that recent DFT works on Pr_4_Ni_3_O_8_ applied the Coulomb repulsion values *U* in different orbital states to purposely remove the contamination from the Pr-4*f* states, which return an almost identical 3*d* band structure near the Fermi level compared to our result from La_4_Ni_3_O_8_ (see fig. S6 for the comparison of the DFT results) (*25*, *26*).

**Fig. 2.**
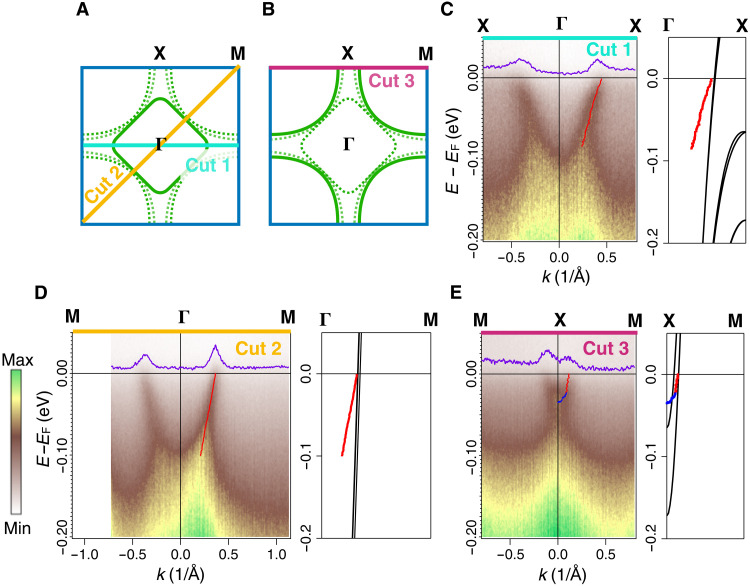
High-symmetry cuts of different pieces of Fermi surface of Pr_4_Ni_3_O_8_. (**A** and **B**) Schematics of Fermi surface. We use 84-eV photons to emphasize the electron pocket (A) and 55-eV photons to emphasize the hole pocket (B). (**C** and **D**) High-symmetry cuts taken with 84-eV photons and (**E**) 55-eV photons. All spectra in this figure are taken at temperature *T* = 22 K. The purple lines in (C) and (D) are the MDCs at the Fermi energy. Band dispersions extracted from the peak positions of MDCs (red dots) and EDCs (blue dots) are plotted in the spectra. To the right of each spectrum, we compare the DFT bands (black curves) of La_4_Ni_3_O_8_ to the MDC and EDC dispersions extracted from the ARPES data.

Figure 2 presents another aspect of the electronic structure in which we show the ARPES dispersions along the high-symmetry cuts. Figure 2 (A and B) denotes the position of the high-symmetry cuts, while the solid (dashed) curves indicate the parts of the Fermi surface that are emphasized (suppressed) by the photon energy choices. The red and blue dots plotted in Fig. 2 (C to E) are the spectral peak positions extracted from momentum distribution curves (MDCs) and energy distribution curves (EDCs), respectively. These spectral peak positions quantify the dispersion in the spectra. Cuts 1 and 2 represent the high-symmetry cuts taken through the electron pocket shown in Fig. 2A in which we observe sharp dispersion down to about 0.1 eV below the Fermi level. In the panels to the right of each spectrum, extracted dispersions are compared to the DFT bands. The chemical potential of the DFT result has been rigidly shifted up by 10 meV to match the experimental result but without any other modifications made. This small chemical potential shift points to an electron count that is slightly away from *d*^8.67^. The actual hole doping level after the 10 meV band shift is 33.8% compared to the 33.3% nominal doping level. This small deviation may arise from imperfect stoichiometry of the sample. With this simple rigid shifting, both cuts 1 and 2 display an excellent match to the Fermi momentum (*k*_F_) of the DFT band. The X-M cut in Fig. 2E is along the so-called “antinodal cut” in hole-doped cuprates, which shows a single band with the shallow band bottom at around 25 meV, qualitatively similar to that in the hole-doped cuprates, where the bottom of the flat bands ranges from about 100 meV below *E*_F_ to just above *E*_F_ (for the antibonding band of the heaviest hole-doped cuprates) (*27*). In the DFT calculations, there are two distinct DFT bands crossing the Fermi level along the X-M direction, while our ARPES measurement only identifies one band and it matches well in *k*_F_ to the DFT band that disperses down to higher binding energy. The extra DFT band in Fig. 2E is part of the invisible hole pocket that we mentioned in the context of Fig. 1E. In the calculations, this extra band predominantly originates from the outer Ni-O plane in the trilayer structure, whereas the two Fermi pockets that match the ARPES observations have a mixed weighting from both the inner and the outer Ni-O plane (see fig. S7). One possible explanation of this discrepancy between DFT and the ARPES observation is the matrix element effect. The matrix element depends on a combination of photon energies, polarizations, experimental geometries, and the sample cleavage plane, all of which can influence the spectral intensity on certain parts of the electronic structure. The total parameter space of the matrix element is hard to exhaust. To explore the matrix element effect, we show a photon energy scan along the X-M cut (cut 3) in fig. S3 and multiple Fermi surface maps taken with different combinations of photon energies, polarizations, and experimental geometries in fig. S10. However, we found no clear evidence of an extra splitting of the hole band. Another possibility is that the broadening of the ARPES dispersions hinder our observation, as the spectral linewidth is larger than this weak band splitting predicted by DFT (see detailed discussions in Supplementary Text S11).

The polarization dependence of our ARPES results (part of the matrix element effects discussed above) indicates that the states which cross the Fermi energy in Pr_4_Ni_3_O_8_ originate from predominantly *d_x_*_^2^__-*y*^2^_ symmetry orbitals—a fact that is confirmed by our DFT calculations (see Supplementary Text S1 and fig. S1 for both the ARPES and DFT results). This finding is consistent with polarization-dependent X-ray absorption measurements reported on these materials (*22*). The dominant *d_x_*_^2^__-*y*^2^_ orbital character of Pr_4_Ni_3_O_8_ is a further critical aspect of the electronic structure analogy between these compounds and the cuprates.

**Fig. 3.**
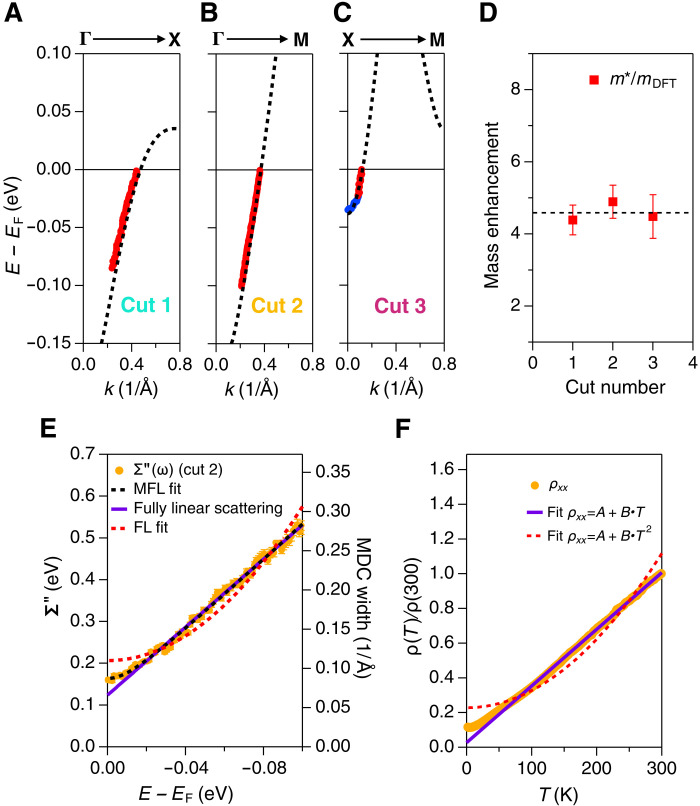
Large effective mass and quasiparticle scattering rate that deviates from the Fermi liquid behavior. (**A** to **C**) MDC (red dots) and EDC (blue dots) dispersions extracted from ARPES spectra in Fig 2 (C to E) and overlaid with the DFT bands renormalized to have a factor of 4 to 5 larger mass without altering the *k*_F_ values. (**D**) Mass enhancement extracted from the ARPES dispersion. The fact that the mass enhancement is approximately *k*-independent indicates that the correlation effects have a local origin, as expected for a Mott-type interaction. (**E**) Extracted imaginary part of the self-energy (proportional to the inverse quasiparticle lifetime) from cut 2. As the band disperses linearly near the Fermi level, the imaginary part of the self-energy can be written as Σ″ = *v*_b_Γ_MDC_/2, where Σ″ is the imaginary part of the self-energy, Γ_MDC_ is the FWHM of the MDCs, and *v*_b_ is the bare band velocity obtained from the DFT band dispersion. The energy dependence of Σ″(ω) shows a much better fit to the MFL model (black dashed curve) than the FL model (red dashed). The standard deviation of the MDC widths from the fitting are included with the data points but are barely visible beyond the point size. (**F**) Resistivity versus temperature. The dashed red line is the fit with a quadratic function, the purple solid line is the linear fit. The resistivity data are taken from (*22*). The vertical bars in (D) represent the errors from extracting the band mass from the ARPES dispersions.

All the DFT bands shown in Fig. 2 disperse to much higher binding energy compared to the ARPES dispersion, indicating a strong mass enhancement effect. We quantify this finding in Fig. 3. Figure 3 (A to C) shows the experimental dispersion (red and blue dots) from Fig. 2 compared to renormalized DFT band dispersions (black dashed lines), where the effective masses of the DFT bands have been renormalized for best agreement to the experimental dispersions (scaling the DFT dispersions to match the ARPES dispersions). The results present overall excellent agreement between the measured and renormalized DFT bands. We note that the DFT renormalization was done while keeping the chemical potential constant at the shifted value, i.e., the renormalization is centered around *E*_F_ as expected for a many-body electronic interaction.

Figure 3D shows the renormalization factors used for the DFT bands in panels (A) to (C), i.e., the effective mass ratio of the ARPES dispersion relative to the corresponding DFT band mass, indicating a strong mass enhancement with values between 4 and 5 for all parts of the Fermi surface (the renormalization factors extracted from Fermi velocities return almost identical results, see fig. S4 and discussions in Supplementary Text S4). These values are approximately a factor of two larger than that of La_4_Ni_3_O_10_ (*16*) and the strange metal state of cuprates, as will be discussed later. This notably large mass enhancement is a signature of a strong electron correlation effect, which is unexpected if, as implied by its nominal hole count, Pr_4_Ni_3_O_8_ can be considered analogous to the heavily hole-doped cuprates. In the cuprates, these heavily doped materials are found to have reduced correlation effects compared to lower-doped compositions that lie nearer to the Mott insulating, strongly correlated regime of the phase diagram (Fig. 1B) (*28*). On the other hand, current many-body calculations have either highly overestimated or underestimated the electronic mass enhancement compared to our experimental observation (*25*, *29*). Our data therefore may be useful for determining why this style of calculation has done a better job for cuprates (*30*) than for the nickelates. In addition, the large mass enhancement in Pr_4_Ni_3_O_8_ is not accompanied by a kink anomaly, such as that found in the superconducting state of cuprates (*31*, *32*), as Fig. 3 (A to C) displays smooth dispersions without any obvious kink features. The kink anomaly in cuprate is a signature of the superconducting state (*31*–*33*). The absence of kink feature in Pr_4_Ni_3_O_8_ may be relevant to the absence of superconductivity. However, we do not exclude the possibility of hidden subtle kink features that would require higher resolution measurements to reveal. Furthermore, the data in Figs. 2 and 3 are taken at *T* = 22 K, slightly higher than the typical *T*_C_ (~15 K) of reported thin film superconducting nickelates (*5*–*9*). Further investigation of Pr_4_Ni_3_O_8_ at lower temperature and the ARPES study of superconducting nickelate compounds are required to identify any signatures of a kink anomaly and to evaluate its potential connection to nickelate superconductivity.

The strong electron correlation in Pr_4_Ni_3_O_8_, signified by the large mass enhancement, also manifests through the quasiparticle scattering rates, which are nominally proportional to the ARPES peak widths. More precisely, we can extract the imaginary part of the electron self-energy function if the spectral dispersion can be approximated by a linear function (see detailed explanation in Supplementary Text S5). In this case, the imaginary self-energy Σ″(ω) is given by (*27*, *28*, *34*)

$$\Sigma^{\prime \prime }(\omega )=v_{B}\frac{\Gamma_{\mathrm{MDC}}(\omega )}{2}$$ (1)

where Γ_MDC_(ω) is the MDC width and *v*_B_ is the bare Fermi velocity, which we obtained from the bare (unrenormalized) DFT band velocity. The orange circles in Fig. 3E show the extracted Σ″(ω) from the “nodal cut” (Γ-M cut or “cut 2,” see fig. S9 for comparison of the MDCs and fits) where the band dispersion is nearly linear. The extracted Σ″(ω) displays an energy dependence that deviates from the quadratic behavior of the conventional Fermi liquid model (red dashed curve), which we write in a generalized form (*28*) as

$$\Sigma_{\mathrm{FL}}^{\prime \prime }(\omega )=\lambda [{\left(\hbar \omega \right)}^{2}+{\left(\beta k_{B}T\right)}^{2}]/\hbar \omega_{N}+\Sigma_{0}^{\prime \prime }$$ (2)

where *T* is the temperature, λ defines the general coupling scale, β is a scaling factor for *k*_B_*T*, Σ_0_^″^ is an offset parameter that accounts for impurity or disorder scattering, and ω_N_ is a normalization frequency that maintains the proper dimensionality of the self-energy (*28*). On the contrary, the marginal Fermi liquid (MFL) model that was used to describe the strange metal states in cuprates presents a good fit to the data. The MFL model was first proposed to explain the non-Fermi liquid behavior found in high-*T*_C_ superconductor cuprates (*35*), which can be written as (*28*)

$$\Sigma_{\mathrm{MFL}}^{\prime \prime }(\omega )=\lambda \sqrt{{\left(\hbar \omega \right)}^{2}+{\left(\beta k_{B}T\right)}^{2}}+\Sigma_{0}^{\prime \prime }$$ (3)

By fitting the data with the parameters λ, β, and $\Sigma_{0}^{\prime \prime }$ (see Supplementary Text S5 for details about the extracted parameters), we get the fit shown by the black dashed lines in Fig. 3E, which trends toward linearity in energy for ω ≫ β*k*_B_*T*, i.e., beyond about 15 meV (purple line), whereas near *E*_F_ the finite temperature leads to some curvature according to Eq. 3. This same form of quasiparticle scattering (imaginary self-energy) is also approximately represented in the resistivity curve versus temperature, although with different proportionality constants that are in general much more complicated to determine (*36*, *37*). Figure 3F plots the normalized resistivity curve ρ(*T*)/ρ(300 K) compared with a quadratic fit (red dashed curve) and a linear fit (solid purple line) (see fig. S12 for measurements from other samples). Similar to the ARPES quasiparticle scattering rate, ρ(*T*) strongly deviates from the quadratic behavior that is expected in the Fermi liquid model. Rather, it presents a strange-metal like behavior, except for a low temperature upturn (<30 K), which is believed to be affected by impurity scattering (*22*). Therefore, the combined behavior of both the ARPES and resistivity data indicates scattering that deviates markedly from standard quadratic Fermi liquid behavior implied by Eq. 2. It is worth noting that the general evolution of the electron scattering from Fermi liquid to non-Fermi liquid behavior can be described by the power law liquid (PLL) model in (*28*).

**Fig. 4.**
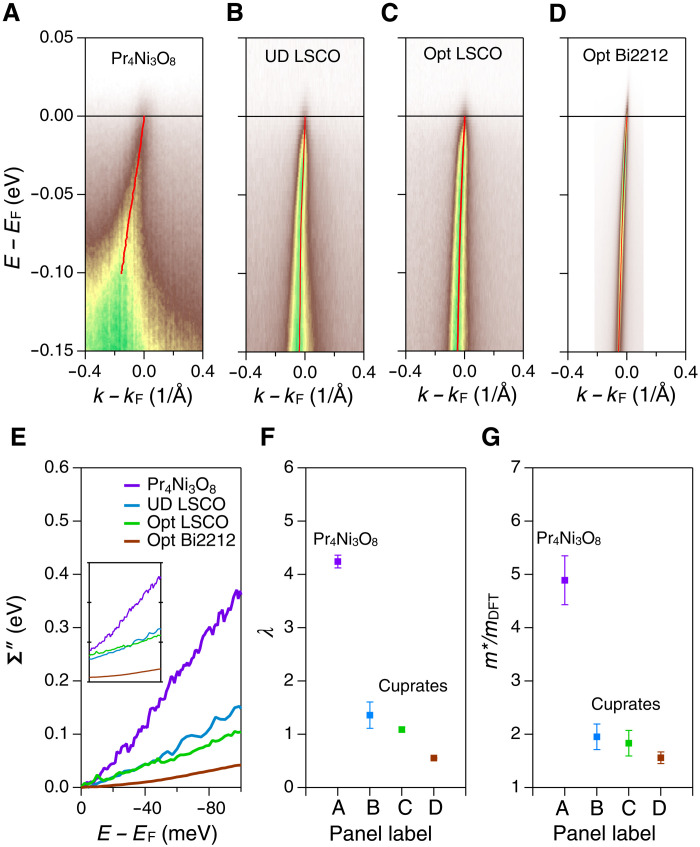
Comparing electronic correlation effects of Pr_4_Ni_3_O_8_ and cuprates. (**A** to **D**) ARPES spectra (cut 2 or nodal cut) of Pr_4_Ni_3_O_8_ in comparison to cuprates. The cuprate samples are underdoped (UD, *x* = 0.08) La_2−*x*_Sr*_x_*CuO_2_ (LSCO), near optimally doped (Opt, *x* = 0.17) LSCO, and optimally doped Bi_2_Sr_2_CaCu_2_O_8+*x*_ (Bi2212). All cuts are taken along (0,0)-(π,π) or the nodal cuts. The red dots denote the MDC peak positions. (**E**) Imaginary self-energy (Σ″) with offset to the value at *E*_F_. The non-offset plot is shown in the inset. (**F**) Slope parameter λ extracted from the Σ″(ω) in (E). The dimensionless coupling constant λ is greater than 4 for the nickelate, whereas those for the cuprates are around 1. (**G**) Mass enhancement extracted from the band dispersions displayed in (A) to (D), showing a two- to threefold larger mass enhancement of nickelate compared to that of cuprates, which we argue is consistent with the increased slope (λ) of (E). The LSCO data are taken at *T* = 25 K (UD) and *T* = 35 K (Opt), whereas the Opt Bi2212 data are taken at *T* = 100 K. All these temperatures are higher than the *T*_C_ of the correspondent samples. The vertical bars in (E) and (F) represent the errors from extracting the slope parameter λ from Σ″(ω) and the band mass from the ARPES dispersions, respectively.

The strange-metal PLL or MFL-like behavior found in Pr_4_Ni_3_O_8_ establishes that its electron dynamics are analogous to that found in the cuprates. Nevertheless, further comparison of the imaginary self-energy in these two types of materials reveals that Pr_4_Ni_3_O_8_ hosts much stronger electronic correlations than the cuprates, consistent with the greater mass enhancements derived from the dispersion data. Figure 4 (A to D) compares the Γ-M or nodal cut of Pr_4_Ni_3_O_8_ (cut 2) to that from three cuprate compounds in the strange metal state above their superconducting transitions. The cuprate spectra display sharper and more coherent peaks, especially in the optimally doped Bi2212, whereas the Pr_4_Ni_3_O_8_ spectrum is markedly broadened toward high binding energies. The Σ″(ω) extracted from MDC widths as in Eq. 1 are plotted in Fig. 4E, quantifying this effect. The Σ″(ω) of Pr_4_Ni_3_O_8_ exhibits a much faster rising slope than the cuprate data, presenting a remarkably stronger dynamic of the electronic interactions. Impurities or defects in crystals can cause broadening of the spectral peak, and we cannot rule out such contributions to the peak width here. However, any such impurity scattering is unrelated to the dynamic trend, i.e., the rising slope of Σ″(ω). Rather, any broadening effect arising from impurities typically appears as a zero-frequency offset in Σ″(ω) and is accounted for by the $\Sigma_{0}^{\prime \prime }$ term in Eq. 3. This zero-frequency offset of Σ″(ω) in Pr_4_Ni_3_O_8_ is similar to what we measured in La_2−*x*_Sr*_x_*CuO_4_ (LSCO; inset of Fig. 4E), which indicates that the Pr_4_Ni_3_O_8_ crystal quality is comparable to the LSCO crystals. The dimensionless coupling constant λ (Eq. 3), extracted from Σ″(ω), describes the intensity of the dynamical excitations and takes the value λ > 4 in Pr_4_Ni_3_O_8_, greatly surpassing the one in cuprates (λ ~ 0.5 to 1.0) as shown in Fig. 4 (*28*). To our knowledge, this is one of the largest energy slopes (or dynamic excitation) found in Σ″(ω) among a variety of well-studied strongly correlated materials, including cuprates, iridates, and ruthenates (*28*, *38*–*40*).

The dynamic excitation in Σ″(ω) is directly connected to the real part of self-energy Σ′(ω) by the Kramers-Kronig relation (*28*, *33*). Electronic mass enhancements, which stem from the Σ′(ω) (see Supplementary Text S6), provide a consistent picture from another perspective. Figure 3D shows that the mass enhancement of the nodal cut (cut 2) of Pr_4_Ni_3_O_8_ is almost 5, where the various cuprate compounds in Fig. 4G show mass enhancements ≤2. Here, we only focused on the comparison to the strange metal state/normal state of the cuprate, which is the precursor state of high-*T*_C_ superconductivity (see Supplementary Text S8 for a more detailed discussion). The substantially larger mass enhancement in Pr_4_Ni_3_O_8_ (over 2.5 times of that in cuprates) is consistent with the larger coupling constant λ (or steeper slope) in Σ″(ω) (see Supplementary Text S6). The comparison to cuprates on both the imaginary self-energy and the mass enhancement indicates that the electronic correlation in Pr_4_Ni_3_O_8_ is of a similar exotic form but substantially stronger than that in cuprates.

## DISCUSSION

Our work provides the first direct observation of the electronic structure in the planar trilayer nickelate, where the Fermi pockets we found are similar to those found in the cuprates, consistent with its *d* orbital occupancy with dominant *d_x_*_^2^__-*y*^2^_ character. The electron correlations found in Pr_4_Ni_3_O_8_ are of a very similar exotic type as in cuprates but are somewhat stronger, as reflected by both the stronger mass enhancement and the larger slope in Σ″(ω).

With this and with other experimental data, we can assemble a type of scorecard of the major physical attributes of the two families of compounds, as shown in Table 1. Row 1 (presence of superconductivity) has already been discussed, although the maximum *T*_C_ of the cuprates remains (for now at least) notably higher. Row 2 shows the result of recent resonant inelastic X-ray scattering (RIXS) measurements indicating the similarity of the magnetic excitations to the 2+ valence cuprates, albeit with a near-neighbor exchange constant that is almost a factor of two smaller (*41*). Rows 3 to 5 are the results directly from this work, indicating the great similarity between the electronic structure and correlations of 1+ valence nickelates compared to 2+ valence cuprates, with the most marked difference being the factor of two increased correlation strength of the nickelates. This increased correlation strength is generally consistent with the concept that the correlation gap of the parent of the nickelates is closer toward the Mott-Hubbard limit (row 6), while the gaps in the cuprates are more charge-transfer like (*3*), although otherwise the similarities between the electronic structure and the correlations are very clear. Last, row 7 asks the question whether the superconductivity in the nickelates should likely have *d_x^2^__-_*_y*^2^*_ pairing symmetry, similar to the cuprates and unlike the vast majority of other superconductors. We argue that it likely should be, not just from the general similarity of the two compounds, but more specifically from the fact that both compounds exhibit a set of flat bands very near (within about 40 meV) *E*_F_ at the (π,0) points of the Brillouin zone. This very high density of states [an extended van Hove singularity (vHs) (*42*)] near *E*_F_ will likely make these states the “drivers” of the superconductivity, while the lower density of states from the zone diagonal will likely make them the “passengers.” We argue that it is therefore likely that a gap of *d_x_*_^2^__-*y*^2^_ symmetry, which derives most of its energy gain from pairing at the (π,0) points and which is generally supported by antiferromagnetic spin fluctuations of the type shown on row 2, should also likely be favored in the nickelate superconductors.

**Table 1.** Scorecard comparing the main physics topics between 2+ valence cuprates and 1+ valence nickelates. For the widest generality, we have mixed the “best” or cleanest results from infinite layer and trilayer nickelates. The similarities between the two columns are overall quite marked although with some numerical differences that may or not be important. AFM, antiferromagnetism; DOS, density of states.

	2+ valence cuprates	1+ valence nickelates	Experimental observation (nickelates)
Superconductivity	to ~160 K	to ~15 K (for now)	D. Li *et al.*. (*5*)
Magnetic excitations	Spin ½ in AFM square lattice. *J*_1_ ~ 110 meV, *J*_2_ ~ −11 meV	Spin ½ in AFM square lattice. *J*_1_ ~ 64 meV, *J*_2_ ~ −10 meV	H. Lu *et al.* (*41*)
Fermi surface shape, geometry, etc.	Extremely similar, including the presence of vHs (flat bands) few tens of meV below *E*_F_ at (π, 0) giving a dominant fourfold DOS contribution. This should support *d*-wave electronic pairing. Additional band accommodates extra hole doping.		This work
Electronic correlations – mass enhancement	About factor of 2 relative to DFT.	About factor of 4 relative to DFT. Consistent with more Mott-like correlations.	This work
Electronic correlations – scattering rate	Anomalously trending toward linearity with *E, T*. Departure from Landau Fermi liquid paradigm. Termed “strange metal,” “marginal Fermi liquid,” etc.	Anomalously trending toward linearity with *E, T*. Departure from Landau Fermi liquid paradigm. The slope is about twice that in cuprates. Consistent with factor of two in mass enhancements.	This work
Mott versus charge transfer gap	More charge-transfer like	More Mott-like. Consistent with stronger correlations.	This work and Goodge *et al.* (*11*)
SC pairing symmetry	*d*_*x*__^2^__− *y*__^2^_	Also *d*_*x*__^2^__−__*y*__^2^_, due to similarity in vHs at (π, 0)?	S. P. Harvey *et al.* (*46*)

Previous works in cuprates have found that the correlation effects in the strange metal phase play a key role for enhancing Cooper pairing (*33*, *43*). Likewise, a recent study on ferropnictides also shows a direct connection between the slope of the scattering rate and *T*_C_ (*39*). In this context, our result raises the important question of whether these stronger strange-metal correlations could support high-temperature superconductivity in Pr_4_Ni_3_O_8_ (*33*, *43*). Nevertheless, while the cuprate lies in the charge-transfer regime of the Zaanen-Sawatzky-Allen scheme, the planar trilayer nickelate, which shows a larger charge transfer gap than that of the cuprate according to a recent RIXS study (*2*), is closer to the Mott-Hubbard regime. The hybridization of *p-d* states in Pr_4_Ni_3_O_8_, however, gives a substantial superexchange coupling *J* of 69 meV, as revealed by another RIXS work (*44*). On the basis of the *t-J* model, the predicted *T*_C_ of the electron-doped planar trilayer nickelate (*23*) would be substantially higher than the maximum *T*_C_ (15 K) of the infinite-layer nickelate (*5*) and potentially on par with optimally doped cuprates. The strong electron correlation we find in Pr_4_Ni_3_O_8_ places these studies on a firmer experimental footing.

In summary, the massive strange-metal correlations in Pr_4_Ni_3_O_8_ provide a platform to explore strange metal physics and its connection to high-temperature superconductivity. More generally, this distinct character to the “overdoped” regime of planar nickelates may serve as a bridge from our well-established descriptions of cuprates to our nascent understanding of the nickelate superconductors.

## MATERIALS AND METHODS

### ARPES measurements

ARPES experiments were carried out on in situ cleaved single crystal surfaces at the Stanford Synchrotron Radiation Lightsource (SSRL) beamline 5-2 and Diamond Lightsource beamline I05. The ARPES data shown in the main text are measured at SSRL beamline 5-2 with an energy resolution of 20 meV. Data in fig. S2 are measured at Diamond beamline I05 with an energy resolution of 5 meV. All data shown here were measured with the photon energy of either 55 or 84 eV unless otherwise noted. All Fermi surface maps shown here are integrated intensity over *E* ± 6 meV.

### Single-crystal growth and transport measurement

Pr_4_Ni_3_O_8_ single crystals (1 to 2 mm^2^ by 0.5 mm) were obtained by reducing specimens cleaved from boules of Pr_4_Ni_3_O_10_ (flowing at 4% H_2_/Ar gas, 350°C, 5 days). High-pressure single-crystal growth of Pr_4_Ni_3_O_10_ was performed in an optical-image floating zone furnace (HKZ-1, SciDre GmbH) with a 140-bar O_2_ (*15*). The crystal structure was identified using X-ray diffraction (*22*). Resistivity of Pr_4_Ni_3_O_8_ single crystals (Fig. 3F) was measured on a Quantum Design Physical Property Measurement System (PPMS) in the temperature range of 2 to 300 K using a conventional four-probe method with contacts made with silver paint.

### DFT calculation

The first principles calculations of La_4_Ni_3_O_8_ and Pr_4_Ni_3_O_8_ (result shown in fig. S5) was performed with the projector-augmented wave method, as implemented in the Vienna Ab Initio Simulation Package. The plane-wave cutoff energy was taken as 400 eV. A full relaxation of the structure was carried out up to the breaking conditions that the total energy change between two electronic steps being less than 1 × 10^−5^ eV, and all forces being smaller than 1 × 10^−3^ eV/Å. The Perdew-Burke-Ernzerhof generalized gradient approximation (GGA) was used to calculate the electronic structure, with a *k*-mesh of 25 × 25 × 9.

The Pr_4_Ni_3_O_8_ DFT band structure at the Fermi level is dominated by the Pr-4*f* states, intersecting the Ni 3*d* bands (see fig. S5). This is inconsistent with our experimental observations. If one includes the effects of electron correlation and applies different Coulomb repulsion values *U* to different orbital states, then one can purposely move the 4*f* states away from *E*_F_ and achieve a 3*d* band structure with similar Fermi surface and bandwidth to that of La_4_Ni_3_O_8_ (*25*, *26*). However, in this work, we need a DFT bare band structure (no electron correlation) as a reference to extract the many-body information from the ARPES results, and the *s*, *p*, and *d* bands of Pr_4_Ni_3_O_8 _and La_4_Ni_3_O_8_ are extremely similar. Thus, we choose to use the bare band structure of La_4_Ni_3_O_8_ in this work.

## Supplementary Materials

### This PDF file includes:

Text S1 to S11 Tables S1 and S2 Figs. S1 to S12 References
