# Supplementary material for: Electronic structure and correlations in planar trilayer nickelate Pr4Ni3O8
Source: Sci Adv. 2023 Jan 13;9(2):eade4418. doi: 10.1126/sciadv.ade4418 (PMC9839319; doi:10.1126/sciadv.ade4418)
Supplement: Supplementary file 1 — Text S1 to S11 Tables S1 and S2 Figs. S1 to S12 References [file sciadv.ade4418_sm.pdf]

Science Advances
AAAS

Supplementary Materials for

Electronic structure and correlations in planar trilayer nickelate $Pr_4Ni_3O_8$

Haoxiang Li *et al.*

Corresponding author: D. S. Dessau, dessau@colorado.edu; J. F. Mitchell, mitchell@anl.gov;
Haoxiang Li, haoxiangli@ust.hk

Sci. Adv. **9**, eade4418 (2023)
DOI: 10.1126/sciadv.ade4418

This PDF file includes:

Text S1 to S11
Tables S1 and S2
Figs. S1 to S12
References

Supplementary Text

S1. Polarization-dependent ARPES Measurement and Orbital Characters

The photon polarization in the ARPES measurement can be used to probe the orbital characters that host different parities with respect to the sample mirror plane. We performed polarization-dependent ARPES measurements to identify the orbital character at the Fermi surface of Pr438. Panel a-b in Fig. S1 shows the Fermi surface of the electron pocket measured by two different photon polarizations. The sample mirror plane is perpendicular to the Fermi surface and lies along the high symmetry direction of Γ-M, indicated by the red and blue lines in Fig. S1 a-b. The p polarization is parallel to the sample mirror plane along the Γ-M direction, and the s polarization is perpendicular to the Γ-M mirror plane. Along the mirror plane, the spectral intensity on the Fermi surface is stronger in the s polarization (panel b) compared to the p polarization (panel a). This contrast can be easily observed by comparison of the two momentum distribution curves (MDC) along the blue and red line in the Fermi surface plot (Fig. S1c). The peak-to -background ratio of the s-polarization MDC curve (blue) is much higher than that of p-polarization (red). Thus, from our ARPES observation, the matrix element effect along the Γ-M direction is emphasized by the s polarization but suppressed by p polarization. Table S1 reveals the corresponding matrix element effect of the dz^2 and dx^2-y^2 orbital. The in-plane dx^2-y^2 orbital has odd parity with respect to the Γ-M mirror plane and it is emphasized by the s-polarization, but suppressed by the p polarization. This is consistent with our observation from ARPES. The outof-plane dz^2 orbital has even parity with respect to the Γ-M mirror plane and thus has an opposite trend, which is in contradiction to our data. The DFT calculation also displays a dominant dx^2-y^2 orbital weighting for the conduction bands (Fig. S1 d-e), which corroborates our ARPES result. Thus, our findings confirm that the dominant orbital character on the Fermi surface is the dx^2-y^2 orbital, which is

consistent with the findings from cuprates and with the previous polarized X-ray absorption spectroscopy study on $Pr_4Ni_3O_8$ (*22*).

	s pol. (odd)	p pol. (even)
dx^2-y^2 (odd)	Allowed	Forbidden
dz^2 (even)	Forbidden	Allowed

Table S1| The matrix elements of states with dx^2-y^2 and dz^2 orbital character along the Γ-M mirror plane.

S2. Gapless Fermi surface

Figure S2 presents the k_F EDCs (panel b) and the correspondent symmetrized EDCs (panel c) taken with high energy resolution (ΔE<5 meV) at different parts of the Fermi surface (positions indicated in panel a). All EDC edges are well aligned with the Fermi level and no obvious gap feature can be resolved from the symmetrized EDCs. Thus, in contrast to the $La_4Ni_3O_{10}$ that displays temperature-dependent energy gaps on the dz^2 orbital band (*16*), the $Pr_4Ni_3O_8$ hosts a gapless Fermi surface with dominant dx^2-y^2 orbital polarization.

S3. Photon Energy Scan

To search for the extra band splitting in the DFT result, we performed a photon energy scan along the antinodal cut (X-M cut, Fig. S3a) where the band splitting is maximized. Fig. S3b shows the Fermi surface along k_z and k_x direction (photon energy ranging from 30 to 130eV). There are three

high intensity peaks centered at $k_x=0$, which can be clearly resolved from the spectral weight integrated along the k_x direction (Fig.3c). The separation between these peaks along k_z matches well with the $2*k_c$ periodicity where $kc=2\pi/c$ (c=25.5Å is the out of plane lattice constant). However, the k_F separation is very small compared to the broadening of the spectral peak, and thus, our data cannot clearly resolve the extra band splitting.

S4. Fermi Velocity Renormalization

The ratio of Fermi velocity between the DFT bands and the ARPES dispersions can also reflect the mass enhancement effect. Consider a parabolic dispersion:

$$E(k) = \frac{\hbar^2 k^2}{2m^*} + E_0 \tag{S1}$$

The Fermi velocity is the first derivative of the band dispersion at the Fermi momentum k_F :

$$v_F = \frac{dE(k)}{dk}|_{k=k_F} = \frac{\hbar^2 k_F}{m^*} \tag{S2}$$

As k_F of the ARPES dispersions and the DFT bands are well aligned (see Fig. 3a-c in the main text), the mass enhancements with respect to the DFT band is then equal to the inverse ratio of the Fermi velocities: $m^*/m^*_{DFT}=v_{DFT}/v_F$. To extract the Fermi velocity, we fit the ARPES dispersion with a linear function within the first 20meV below the Fermi level. The fit results are shown in Fig. S4a-c. Fig. S4d shows the Fermi velocity renormalization, which is the ratio of Fermi velocity between ARPES dispersions and DFT bands. Compared to the effective mass shown in the main text, the Fermi velocity renormalization exhibits almost identical results.

S5. Spectral Function and Self-energy

ARPES is a direct measurement of the electron spectral function. which can in general be written

as:

$$A(k,\omega) = -\frac{1}{\pi}\frac{\Sigma''}{(\omega-\epsilon_k-\Sigma')^2+\Sigma''^2} \tag{S3}$$

where Σ' and Σ'' are the real and imaginary parts of the electron self-energy, and ϵ_k is the bare band dispersion. With the assumption of a linear bare dispersion $\epsilon_k = v_b(k - k_F)$, a reasonable approximation when the band bottom/band top are far from the Fermi level, the spectral function can then be expressed as:

$$A(k,\omega) = -\frac{1}{\pi v_b}\frac{\Sigma''/v_b}{[k-(k_F+\frac{\Sigma'-\omega}{v_b})]^2+[\Sigma''/v_b]^2} \tag{S4}$$

For a Momentum Distribution Curve (MDC) of constant energy ω, the spectral function can be rewritten as a Lorentzian functional form:

$$A(k) = \frac{I}{\pi}\frac{\Gamma/2}{(k-k_0)^2+(\Gamma/2)^2} \tag{S5}$$

where Γ is the full width half maximum (FWHM) of the MDC. This shows that the imaginary part of the self-energy Σ'' is directly proportional to FWHM of MDC:

$$\Sigma''(\omega) = v_b \times \Gamma/2 \tag{S5}$$

It is worth noting that, in Fig. S4 b, the ARPES dispersion of cut 2 displays a nice fit to the linear function, even though the linear fit is constrained to the first 20 meV. Thus, the dispersion of cut 2 is an approximately linear dispersion, and we can use the method discussed above to extract the imaginary self-energy $\Sigma''(\omega)$ of cut 2, which is the ARPES cut in the main text for which we present the detailed analysis of $\Sigma''(\omega)$.

In Fig. 3E of the main text, we fit the $\Sigma''(\omega)$ from ARPES to the marginal Fermi liquid model described in Eqn. 3 of the main text. The extracted parameter reflecting the rising slope of $\Sigma''(\omega)$

is λ= 4.2±0.1, whereas similar parameters extracted from multiple dopings of Bi2212 is only at 0.5 according to our data and previous study (*28*). The temperature scaling factor β=7.3±0.8, and $\beta k_B T$=14 meV, where k_B is the Boltzmann constant, T=22 K is data-taking temperature. Thus, beyond about 15meV, the $\Sigma''(\omega)$ present a nearly linear energy dependence.

S6. Self-energy (Σ' and Σ'') and mass enhancement

Here we show how the real part of the self-energy renormalizes the band dispersion by adding Σ' to the bare band term. The spectral function in Eqn. (S3) can be rewritten in terms of the renormalization factor Z as:

$$A(k,\omega) = -\frac{1}{\pi}\frac{\Sigma''}{Z^2(\omega-\frac{\epsilon_k}{Z})^2+\Sigma''^2} \tag{S6}$$

The pole of the spectral function is $\omega=\epsilon_k/Z$, where Z=1-Σ'/ω denotes the renormalization to the bare band dispersion ϵ_k. This is also the expression of the mass enhancement with respect to the bare band:

$$\frac{m^*}{m_{bare}} = Z = 1-\frac{\Sigma'(\omega)}{\omega} \tag{S7}$$

The mass enhancement is thus directly proportional to the real part of the self-energy Σ'. On the other hand, the real and imaginary part of the self-energy follow the Kramers-Kronig relation:

$$\Sigma'(\omega) = -\frac{1}{\pi}\int_{-\omega_c}^{\omega_c}\frac{\Sigma''(\omega')}{\omega'-\omega}d\omega' = -\frac{1}{\pi}\int_{-\omega_c}^{\omega_c}\frac{\lambda\omega'}{\omega'-\omega}d\omega' \tag{S8}$$

here we assume $\Sigma''(\omega')=\lambda\omega'$ consistent with the marginal Fermi liquid model at zero temperature, and ω_c is the cut-off energy. Although the exact solution of this integral depends on the cut-off energy (typically considered to be around the bare band bottom energy), one can tell that the real part of the self-energy Σ' is proportional to the linear slope λ in Σ'', with the larger λ naturally

corresponding to stronger band renormalization, and thus, larger mass enhancement (Eqn. S7). Therefore, in Fig. 4 of the main text, the larger slope of Σ'' in $Pr_4Ni_3O_8$ corroborates with the larger effective mass compared to other cuprate samples, even though these two properties of the many-body system—i.e., the quasiparticle scattering rate and the mass renormalization, are extracted from different aspects of the ARPES spectra (MDC widths and dispersion).

S7. DFT result of $Pr_4Ni_3O_8$

The electronic structure of $Pr_4Ni_3O_8$ was calculated using a conventional I4/mmm tetragonal unit cell with lattice constants a=3.9347Å, c=25.485Å, using the same method as described for $La_4Ni_3O_8$ in the main paper. Fig. S5 shows the calculated band structure of $Pr_4Ni_3O_8$, where a concentration of heavy (flat) bands appears around E_F that originate from the 4f electrons of Pr ions. This large number of flat bands complicates a direct comparison of the band structure with the ARPES data. We found, however, that the calculated *s*, *p*, and *d* bands of $Pr_4Ni_3O_8$ and $La_4Ni_3O_8$ are extremely similar - thus, we replace Pr atoms with La to avoid the contamination from the Pr 4*f* states. In another DFT work (*25*), the contamination of Pr 4*f* states at the Fermi level is removed by tweaking the Coulomb repulsion U. As shown in Fig. S6, the conduction band structure of $Pr_4Ni_3O_8$ (with the 4*f* states removed by U) is almost identical to that of La438. This further justify our comparison to the DFT result of La438 in the main text.

S8. Electron occupancy at the two difference Ni sites

Figure S7 shows the electron occupancy at the two difference Ni sites at the inner NiO plane and the outer NiO plane. The extra band that was not picked up by our ARPES measurement shows predominant weighting from the outer NiO plane, whereas the other two bands that cross the Fermi level display mixture weighting from both inner and outer planes. This result can also be observed in the Fermi surface occupation. As shown in Fig. S8. the missing piece of the Fermi surface from ARPES is mainly contributed by the outer Ni site, whereas the other two pieces of the Fermi

surface present mixtures of both Ni sites. The oxygen *p* states actually have finite occupancy at the Fermi level even though it's very small (<10%).

S9. Mass enhancement of cuprates

The mass enhancements of the cuprates shown in the main text are consistent with previous studies in the normal state strange-metal phase (*31,32,33,47,48*), which showed that the normal-state antinodal mass enhancement has a similar value (*47*). Below the superconducting transition temperature, however, the antinodal effective mass of cuprates is enlarged to a value ~7 due to an additional strong low-energy renormalization or "kink" features (*33,47*); $Pr_4Ni_3O_8$ does not show signs of superconductivity and displays a smooth dispersion that shows no sign of a kink anomaly. However, we do not exclude the possibility that higher resolution measurement in future studies may reveal hidden subtle feature in the dispersion. We also note that certain cuprate compounds display higher effective masses at the quantum limit under extreme conditions of low temperature and high external magnetic field (*49,50*). In the main text, we only focused on the comparison to the strange metal state/normal state of the cuprate, which is the major precursor state of high T_C superconductivity.

S10. Resistivity of $Pr_4Ni_3O_8$

The absolute value of resistivity at room temperature is relatively large (on the scale of Ω cm). Table S2 below shows the absolute resistivity of $Pr_4Ni_3O_8$ together with a few La doped variants where less metallicity is expected as the La content is increased (*51*). However, the absolute values of resistivity are all on the scale of Ω cm and show no trend towards metallicity on the Pr rich side. The resistance in these samples is probably dominated by extrinsic contribution, likely from the microcracks in the crystals, as unavoidable cracking occurs during the topochemical reduction

process. As the $Pr_4Ni_3O_8$ samples are fragile after the reduction process, it is difficult to get a large area of nicely cleaved surface on this material. However, with the small size (~10 μm) beam spot at the synchrotron based ARPES beamlines (SSRL 5-2 and Diamond I05), we were able to locate tiny, good emission spots on the cleaved surface.

$(Pr_{1-x}La_x)_4Ni_3O_8$	x=0	x=0.1	x=0.3	x=0.4	x=0.5	x=0.7	x=0.9
Resistivity at 300 K (Ω cm)	3.3<br>0.97	5.83	10.45	1.24	2.08	10.62	3.48

Table S2. Measured resistivity at 300 K for specimens of parent $Pr_4Ni_3O_8$ and La doped variants. Data for x=0 (Sample #2 and Sample #3) are shown in Fig. S12. They of comparable magnitude with no systematic doping dependence. It is likely that the extrinsic contribution (possibly from microcracks) dominates the resistance at all temperatures and thus mask the potential trend towards metallicity on the Pr rich side of the phase diagram.

S11. Spectral linewidth and DFT band splitting

The exact linewidth at E_F of $Pr_4Ni_3O_8$ is ~0.12 $Å^{-1}$ (FWHM of MDC in Fig. 2D). The largest band splitting from the DFT calculation is ~0.05 $Å^{-1}$ (at E_F), which is smaller than the spectral linewidth and potentially make it difficult to resolve. However, the spectral linewidth of $Pr_4Ni_3O_8$ at the Fermi level is not large, and it is comparable to the linewidth of the cuprate samples (see Fig. 4). In the inset of Fig. 4E, we compare the raw Σ'' of the $Pr_4Ni_3O_8$ and the cuprate samples. One can see that the impurity scattering rate (scattering rate at the Fermi level) of $Pr_4Ni_3O_8$ is very close to that of the $La_{2-x}Sr_xCuO_2$ samples. Although the broadening from the impurity scattering is

relatively low, the dramatic increase of the scattering rate due to the dynamic effect (the rising slope of Σ'' in energy) certainly lead to a heavily broadened band feature and hinder the observation of two band that is close to degenerate.

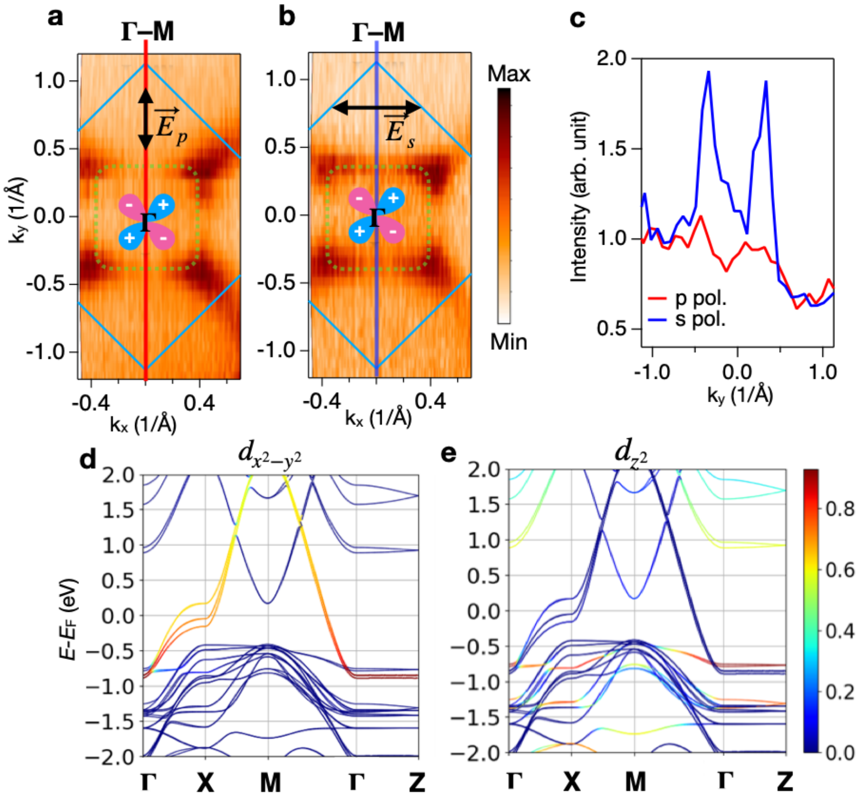

Fig. S1. Polarization dependent ARPES measurements of $Pr_4Ni_3O_8$. (**a-b**) Fermi surface measured with two different linear polarized 84 eV photon beams, the light blue box represents the BZ boundary, the dashed green line outlines the Fermi surface. The photon incidence plane is aligned with the **Γ**-M mirror plane of the sample, which is labeled by the red and blue line in panel **a** and **b**. The dx^2-y^2 orbital is odd with respect to the **Γ**-M mirror plane, thus, the odd symmetry s polarization provides favorable matrix elements, whereas the even symmetry p polarization is unfavored (see text). Along **Γ**-M (the red and blue vertical lines), the Fermi surface is disconnected in panel **a** (p pol.), but shows a continuing feature in panel **b** (s pol.).(**c**) Momentum distribution cut (MDC) at -20meV along the red and blue lines of panels **a** & **b** (**d, e**) The DFT band structure

of $La_4Ni_3O_8$ with the orbital weighting indicated by the color scale. The out-of-plane dz^2 orbital is even with respect to the **Γ**-M mirror plane, which means the polarization effect should be opposite to the dx^2-y^2 orbital.

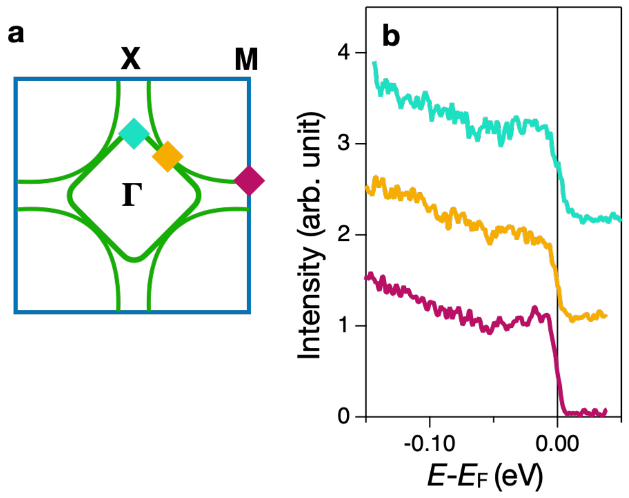

Fig. S2. The non-gapped Fermi surface measured with high resolution ARPES. (**a**) The schematics of Fermi surface in which the colored dots indicate the position of the k_F EDCs shown in panel (**b**). (**c**) The symmetrized k_F EDCs as shown in the panel (**b**). The data in this figure are taken at low temperature (~5K) with high energy resolution (<5meV).

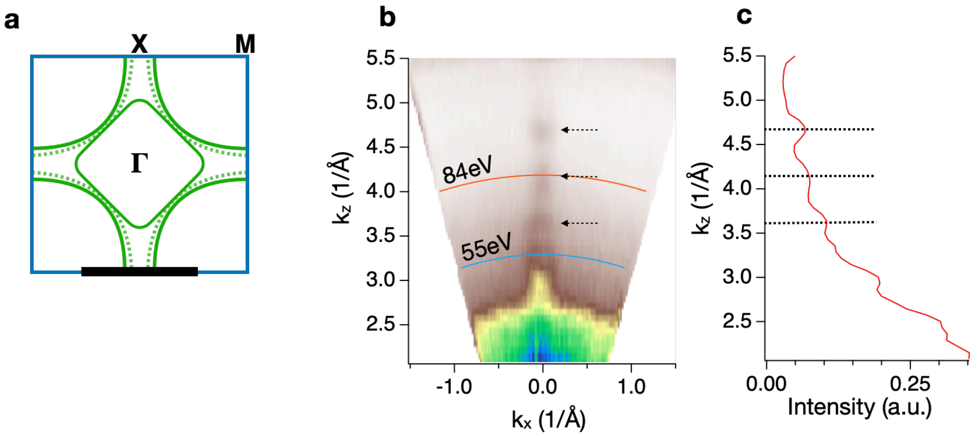

Fig. S3. photon energy scan along X-M cut. (**a**) Schematic drawing of the in-plane Fermi surface. The black line indicate the cut position in the in-plane Brillouin Zone. The photon energy scan cut is taken along (-**π**, **π**)-(**π**,**π**) direction. (**b**) Fermi surface along k_z and k_x direction. The two color curves indicate the cuts with 55eV and 84eV photon energy (**c**) The spectral weight of the Fermi

surface integrated along the k_x direction, where 3 intensity peaks can be well resolved that follow the k_z periodicity (see discussion in Supplementary Text S3).

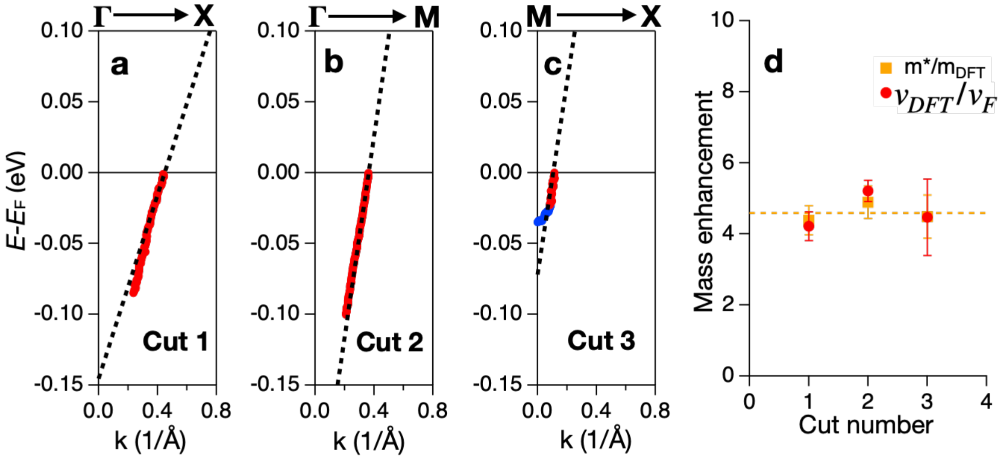

Fig. S4. **Fermi velocity renormalization.** (**a-c**) ARPES dispersion and the linear fit to the first 20meV below Fermi level to extract the Fermi velocity. The MDC (red dots) and EDC (blue dots) dispersions are extracted from spectra in Fig 2 C-E and overlaid with the linear fit (black dashed curved). (**d**) The enhancement relative to the DFT band extracted from first derivatives (Fermi velocity v_F) and second derivatives (mass enhancement shown in the main text).

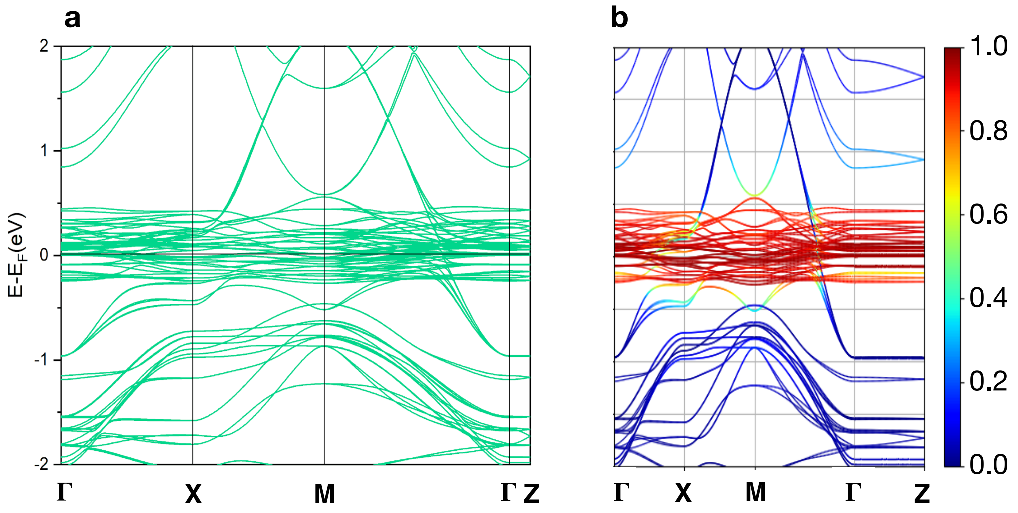

Fig. S5. DFT results of $Pr_4Ni_3O_8$. (**a**) The calculated band structure of $Pr_4Ni_3O_8$ (calculations for $La_4Ni_3O_8$ were shown in the main paper); (**b**) Projected 4*f*-bands, with the color scale indicating the corresponding contribution of 4*f* electrons to certain band areas.

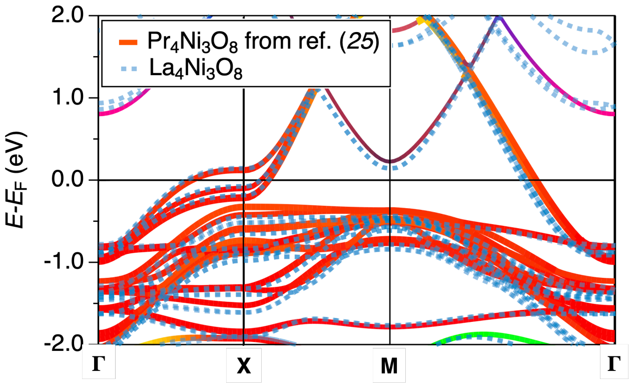

Fig. S6. Compare DFT band structure of $La_4Ni_3O_8$ and $Pr_4Ni_3O_8$ from ref. (*25*). The DFT band structure of $Pr_4Ni_3O_8$ is extracted from Fig. 2d in ref. (*25*). The $La_4Ni_3O_8$ band structure is basically identical to the $Pr_4Ni_3O_8$ one, which tweak the Coulomb repulsion U to remove the contamination of Pr 4f states.

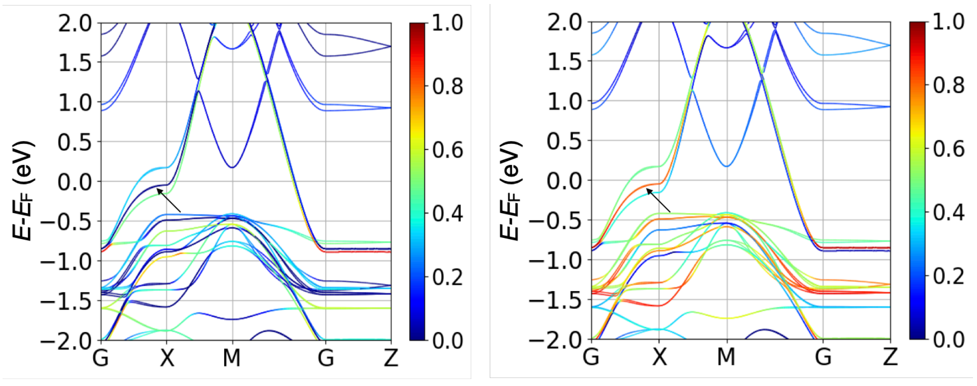

Fig. S7. Band occupancy at Ni sites from the inner and outer planes. The color scale in the figure represents the occupancy of electrons from the inner Ni-O plane (left panel) and the outer Ni-O plane (right panel). The extra band that is missing from the ARPES measurement (pointed out by the black arrow) is predominantly from the outer plane, whereas the other two bands crossing the Fermi level shows a mixture from both inner and outer planes.

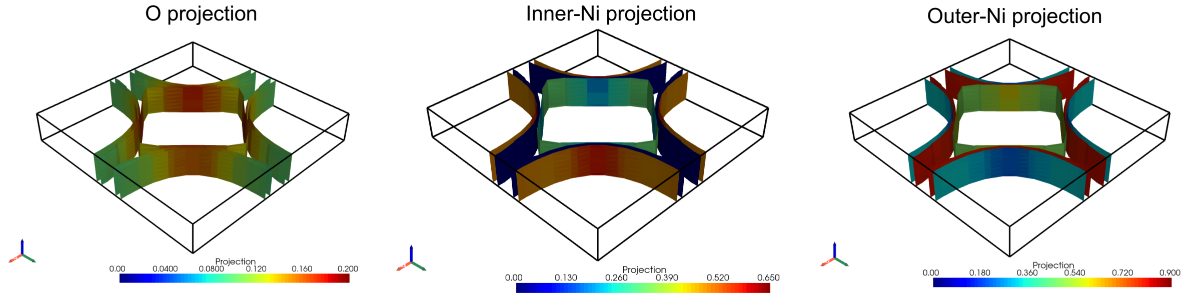

Fig. S8. Fermi surface occupancy. The Fermi surface occupancies of oxygen (left panel) and two different Ni sites from inner Ni-O plane (middle panel) and outer Ni-O plane (right panel) are presented

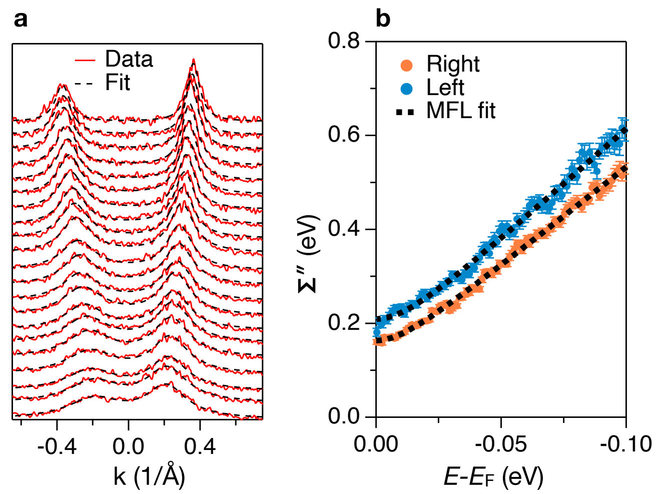

Fig. S9. Lorentzian fitting to MDCs. Panel **a** plot the stacking MDCs (red line) of the nodal cut (cut 2) shown in Fig. 2D in the main text, and the fit to double Lorentzian (dashed black line). The MDC widths are extracted from the Lorentzian fitting. Panel **b** shows the extracted self-energy Σ″ that is directly proportional to MDC widths of the left and the right bands. Both curves show a good fit to the MFL model (dashed black curve) and return with a consistent coupling constant λ of 4.2 (right) and 4.7 (left). In the main text, we present the result from the right band as it shows a dominant peak intensity due to the experiment geometry.

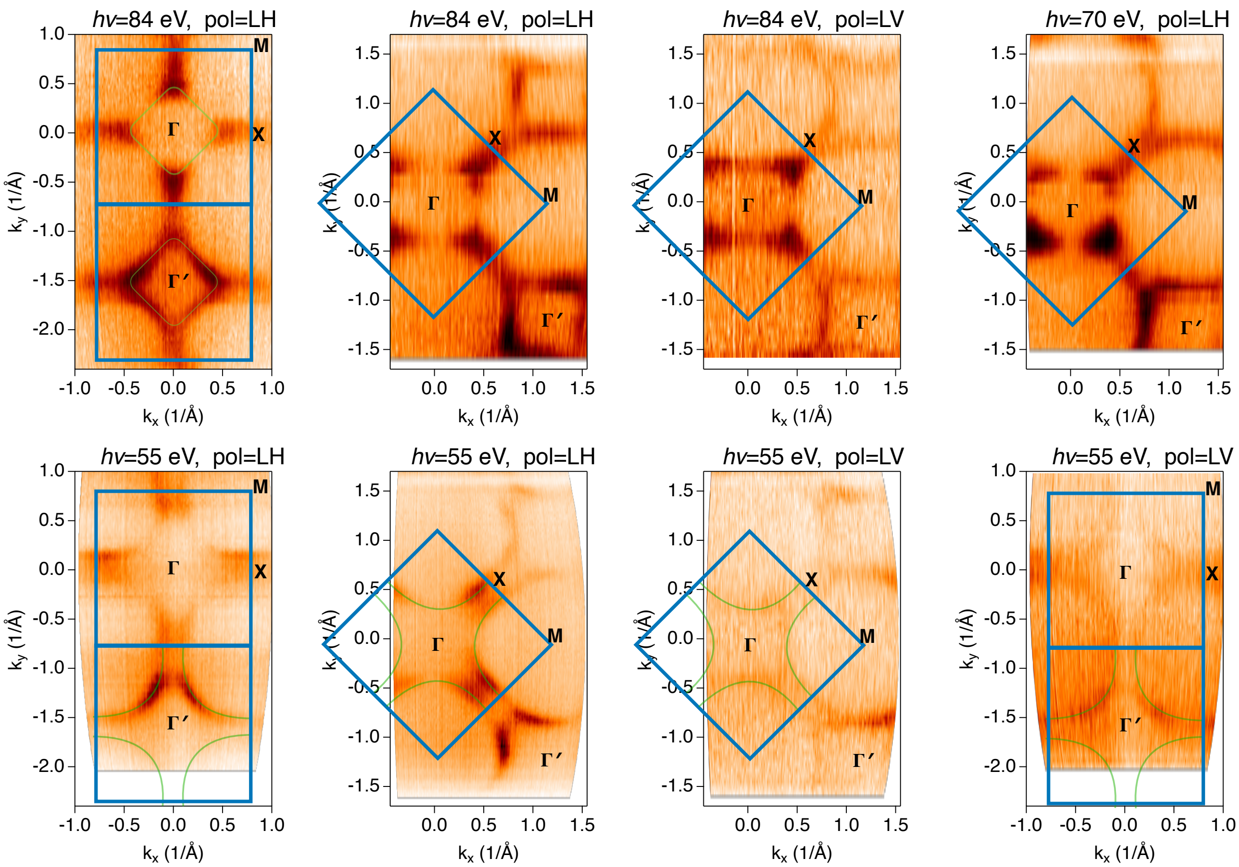

Fig. S10. Fermi surface maps taken with different combinations of photon energies, photon polarizations and experimental geometries. The linear horizontal and linear vertical photon polarization are labeled as LH and LV, respectively. One can find that although the polarization and experimental geometry cause the variation of spectral intensity to some degree, Fermi surface maps taken at 84 eV only display the electron pocket centered around Γ, whereas the 55 eV photon reveals the same hole pocket regardless of the experimental geometry and polarization. The 70eV photon also reveal the same electron pocket as shown in 84 eV photon data. Thus, for simplification, we used the photon energy to categorize the data in the main text.

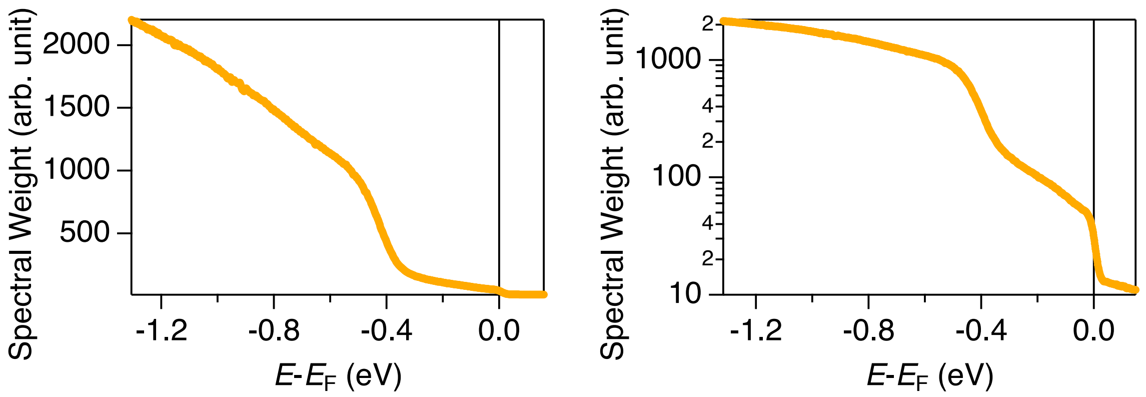

Fig. S11. Spectral weight plotted in linear scale (left) and log scale (right). At 350 meV below Fermi level, there is a dramatic upturn of the spectral weight and the intensity at 1eV below E_F is over two orders of magnitude larger than the spectral intensity near E_F. This extremely strong spectral intensity is potentially given by the Pr *f* states.

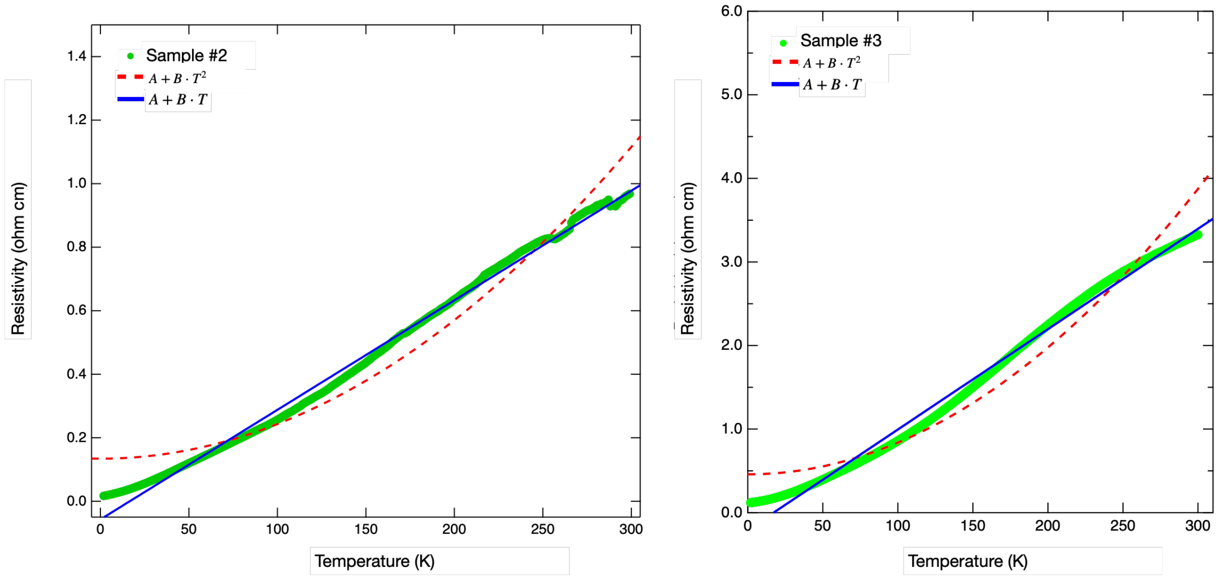

Fig. S12. Resistivity curves of $Pr_4Ni_3O_8$. Although the absolute value of the room temperature resistivity differs between the two samples, the resistivity curves consistently deviate from the quadratic fit but closer to the linear model.

REFERENCES AND NOTES

1. K.-W. Lee W. E. Pickett, Infinite-layer $LaNiO_2$: $Ni^{1+}$is not Cu^{2+}. *Phys. Rev. B* **70**, 165109 (2004).

2. Y. Shen, J. Sears, G. Fabbris, J. Li, J. Pelliciari, I. Jarrige, Xi He, I. Božović, M. Mitrano, J. Zhang, J. F. Mitchell, A. S. Botana, V. Bisogni, M. R. Norman, S. Johnston, M. P. M. Dean, Role of oxygen states in the low valence nickelate $La_4Ni_3O_8$. *Phys. Rev. X* **12**, 011055 (2022).

3. J. Zaanen, G. A. Sawatzky, J. W. Allen, Band gaps and electronic structure of transition-metal compounds. *Phys. Rev. Lett.* **55**, 418–421 (1985).

4. F. C. Zhang, T. M. Rice, Effective Hamiltonian for the superconducting Cu oxides. *Phys. Rev. B* **37**, 3759(R) (1988).

5. D. Li, K. Lee, B. Y. Wang, M. Osada, S. Crossley, H. R. Lee, Y. Cui, Y. Hikita, H. Y. Hwang, Superconductivity in an infinite-layer nickelate. *Nature* **572**, 624–627 (2019).

6. M. Osada, B. Y. Wang, B. H. Goodge, K. Lee, H. Yoon, K. Sakuma, D. Li, M. Miura, L. F. Kourkoutis, H. Y. Hwang, A superconducting praseodymium nickelate with infinite layer structure. *Nano Lett.* **20**, 5135–5740 (2020).

7. M. Osada, B. Y. Wang, B. H. Goodge, S. P. Harvey, K. Lee, D. Li, L. F. Kourkoutis, H. Y. Hwang, Nickelate superconductivity without rare-earth magnetism: $(La,Sr)NiO_2$. *Adv. Mater.* **33**, 2104083 (2021.

8. S. Zeng, C. Li, L. E. Chow, Y. Cao, Z. Zhang, C. S. Tang, X. Yin, Z. S. Lim, J. Hu, P. Yang, A. Ariando, Superconductivity in infinite-layer nickelate $La_{1-x}Ca_xNiO_2$ thin films. *Sci. Adv.* **8**, eabl9927 (2022).

9. G. A. Pan, D. F. Segedin, H. LaBollita, Q. Song, E. M. Nica, B. H. Goodge, A. T. Pierce, S. Doyle, S. Novakov, D. C. Carrizales, A. T. N'Diaye, P. Shafer, H. Paik, J. T. Heron, J. A. Mason, A. Yacoby, L. F. Kourkoutis, O. Erten, C. M. Brooks, A. S. Botana, J. A. Mundy,

Superconductivity in a quintuple-layer square-planar nickelate. *Nat. Mater.* **21**, 160–164 (2022).

10. G.-M. Zhang, Y.-F. Yang, and F.-C. Zhang, Self-doped Mott insulator for parent compounds of nickelate superconductors. *Phys. Rev. B* **101**, 020501(R) (2020).

11. B. H. Goodge, D. Li, K. Lee, M. Osada, B. Y. Wang, G. A. Sawatzky, H. Y. Hwang, L. F. Kourkoutis, Doping evolution of the Mott-Hubbard landscape in infinite-layer nickelates. *Proc. Natl. Acad. Sci. U.S.A.* **118**, e2007683118 (2021).

12. Z.-J. Lang, R. Jiang, W. Ku, Strongly correlated doped hole carriers in the superconducting nickelates: Their location, local many-body state, and low-energy effective Hamiltonian *Phys. Rev. B* **103**, L180502 (2021).

13. Y. Nomura, T. Nomoto, M. Hirayama, R. Arita, Magnetic exchange coupling in cuprate-analog d^9 nickelates. *Phys. Rev. Research* **2**, 043144 (2021).

14. J. Krishna, H. LaBollita, A. O. Fumega, V. Pardo, A. S. Botana, Effects of Sr-doping on the electronic and spin-state properties of infinite-layer nickelates. *Phys. Rev. B* **102**, 224506 (2020).

15. J. Zhang, H. Zheng, Y.-S. Chen, Y. Ren, M. Yonemura, A. Huq, J. F. Mitchell, High oxygen pressure floating zone growth and crystal structure of the metallic nickelates $R_4Ni_3O_{10}$ (R = La, Pr). *Phys. Rev. Mater.* **4**, 083402 (2020).

16. H. Li, X. Zhou, T. Nummy, J. Zhang, V. Pardo, W. E. Pickett, J. F. Mitchell, D. S. Dessau, Fermiology and electron dynamics of trilayer nickelate $La_4Ni_3O_{10}$. *Nat. Commun.* **8,** 704 (2017).

17. J. Chaloupka, G. Khaliullin, Orbital order and possible superconductivity in $LaNiO_3/LaMO_3$ superlattices. *Phys. Rev. Lett.* **100**, 016404 (2008).

18. M. Uchida, K. Ishizaka, P. Hansmann, Y. Kaneko, Y. Ishida, X. Yang, R. Kumai, A. Toschi, Y. Onose, R. Arita, K. Held, O. K. Andersen, S. Shin, Y. Tokura, Pseudogap of metallic

layered nickelate $R_{2-x}Sr_xNiO_4$ (R = Nd, Eu) crystals measured using angle-resolved photoemission spectroscopy. *Phys. Rev. Lett.* **106**, 027001 (2011).

19. M. Uchida, Y. Yamasaki, Y. Kaneko, K. Ishizaka, J. Okamoto, H. Nakao, Y. Murakami, Y. Tokura, Pseudogap-related charge dynamics in the layered nickelate $R_{2-x}Sr_xNiO_4$ ($x \sim 1$). *Phys. Rev. B* **86**, 165126 (2012).

20. S. Anissimova, D. Parshall, G. D. Gu, K. Marty, M. D. Lumsden, S. Chi, J. A. Fernandez-Baca, D. L. Abernathy, D. Lamago, J. M. Tranquada, D. Reznik, Direct observation of dynamic charge stripes in $La_{2-x}Sr_xNiO_4$. *Nat. Commun.* **5**, 3467 (2014).

21. H. Y. Hwang, Y. Iwasa, M. Kawasaki, B. Keimer, N. Nagaosa, Y. Tokura, Emergent phenomena at oxide interfaces. *Nat. Mater.* **11**, 103–113 (2012).

22. J. Zhang, A. S. Botana, J. W. Freeland, D. Phelan, H. Zheng, V. Pardo, M. R. Norman, J. F. Mitchell, Large orbital polarization in a metallic square-planar nickelate. *Nat. Phys.* **13**, 864–869 (2017).

23. E. M. Nica, J. Krishna, R. Yu, Q. Si, A. S. Botana, O. Erten, Theoretical investigation of superconductivity in trilayer square-planar nickelates. *Phys. Rev. B* **102**, 020504(R) (2020).

24. H. Sakakibara, H. Usui, K. Suzuki, T. Kotani, H. Aoki, K. Kuroki, Model construction and a possibility of cupratelike pairing in a New d^9 nickelate superconductor(Nd,Sr)NiO_2. *Phys. Rev. Lett.* **125**, 077003 (2020).

25. J. Karp, A. Hampel, M. Zingl, A. S. Botana, H. Park, M. R. Norman, A. J. Millis, A comparitive many-body study of $Pr_4Ni_3O_8$ and $NdNiO_2$. *Phys. Rev. B* **102**, 245130 (2021).

26. A. S. Botana, V. Pardo, M. R. Norman, Electron doped layered nickelates: Spanning the phase diagram of the cuprates. *Phys. Rev. Materials* **1**, 021801(R) (2017).

27. A. Damascelli, Z. Hussain, Z.-X. Shen, Angle-resolved photoemission studies of the cuprate superconductors. *Rev. Mod. Phys.* **75**, 473–541 (2003).

28. T. J. Reber, X. Zhou, N. C. Plumb, S. Parham, J. A. Waugh, Y. Cao, Z. Sun, H. Li, Q. Wang, J. S. Wen, Z. J. Xu, G. Gu, Y. Yoshida, H. Eisaki, G. B. Arnold, D. S. Dessau, A unified form of low-energy nodal electronic interactions in hole-doped cuprate superconductors. *Nat. Commun.* **10**, 5737 (2019).

29. F. Lechermann, Emergent flat-band physics in $d^{9-\delta}$ multilayer nickelates, *Phys. Rev. B* **105**, 155109 (2022).

30. C. Weber, K. Haule, G. Kotliar, Strength of correlations in electron- and hole-doped cuprates. *Nat. Phys.* **6**, 574–578 (2010).

31. A. Lanzara, P. V. Bogdanov, X. J. Zhou, S. A. Kellar, D. L. Feng, E. D. Lu, T. Yoshida, H. Eisaki, A. Fujimori, K. Kishio, J.-I. Shimoyama, T. Noda, S. Uchida, Z. Hussain, Z.-X. Shen, Evidence for ubiquitous strong electron–phonon coupling in high-temperature superconductors. *Nature* **412**, 510–514 (2001).

32. T. Cuk, F. Baumberger, D. H. Lu, N. Ingle, X. J. Zhou, H. Eisaki, N. Kaneko, Z. Hussain, T. P. Devereaux, N. Nagaosa, Z.-X. Shen, Coupling of the B1g phonon to the antinodal electronic states of $Bi_2Sr_2Ca_{0.92}Y_{0.08}Cu_2O_{8+\delta}$. *Phys. Rev. Lett.* **93**, 117003 (2004).

33. H. Li, X. Zhou, S. Parham, T. J. Reber, H. Berger, G. B. Arnold, D. S. Dessau, Coherent organization of electronic correlations as a mechanism to enhance and stabilize high-T_C cuprate superconductivity. *Nat. Commun.* **9**, 26 (2018).

34. T. Valla, A. V. Fedorov, P. D. Johnson, Q. Li, G. D. Gu, N. Koshizuka, Temperature dependent scattering rates at the Fermi surface of optimally doped $Bi_2Sr_2CaCu_2O_{8+\delta}$. *Phys. Rev. Lett.* **85**, 828–831 (2000).

35. C. M. Varma, P. B. Littlewood, S. Schmitt-Rink, E. Abrahams, A. E. Ruckenstein, Phenomenology of the normal state of Cu-O high-temperature superconductors. *Phys. Rev. Lett.* **63**, 1996–1999 (1989).

36. A. Lucas, S. A. Hartnoll, Resistivity bound for hydrodynamic bad metals. *Proc. Natl. Acad. Sci. U.S.A.* **114**, 11344–11349 (2017).

37. A. Lucas, S. A. Hartnoll, Kinetic theory of transport for inhomogeneous electron fluids. *Phys. Rev. B* **97**, 045105 (2018).

38. Y. Cao, Q. Wang, J. A. Waugh, T. J. Reber, H. Li, X. Zhou, S. Parham, S.-R. Park, N. C. Plumb, E. Rotenberg, A. Bostwick, J. D. Denlinger, T. Qi, M. A. Hermele, G. Cao, D. S. Dessau, Hallmarks of the Mott-metal crossover in the hole-doped pseudospin-1/2 Mott insulator Sr_2IrO_4. *Nat. Commun.* **7**, 11367 (2016).

39. J. Fink, E. D. L. Rienks, M. Yao, R. Kurleto, J. Bannies, S. Aswartham, I. Morozov, S. Wurmehl, T. Wolf, F. Hardy, C. Meingast, H. S. Jeevan, J. Maiwald, P. Gegenwart, C. Felser, B. Büchner, Linkage between scattering rates and superconductivity in doped ferropnictides. *Phys. Rev. B* **103**, 155119 (2021).

40. P. Kostic, Y. Okada, N. C. Collins, Z. Schlesinger, J. W. Reiner, L. Klein, A. Kapitulnik, T. H. Geballe, M. R. Beasley, Non-fermi-liquid behavior of $SrRuO_3$: Evidence from infrared conductivity. *Phys. Rev. Lett.* **81**, 2498–2501 (1998).

41. H. Lu, M. Rossi, A. Nag, M. Osada, D. F. Li, K. Lee, B. Y. Wang, M. Garcia-Fernandez, S. Agrestini, Z. X. Shen, E. M. Been, B. Moritz, T. P. Devereaux, J. Zaanen, H. Y. Hwang, K.-J. Zhou, W. S. Lee, Magnetic excitations in infinite-layer nickelates. *Science* **373**, 213–216 (2021).

42. D. S. Dessau, Z.-X. Shen, D. M. King, D. S. Marshall, L. W. Lombardo, P. H. Dickinson, A. G. Loeser, J. DiCarlo, C.-H Park, A. Kapitulnik, W. E. Spicer, Key features in the measured bandstructure of $Bi_2Sr_2CaCu_2O_{8+d}$: Flat bands at E_F and fermi surface nesting. *Phys. Rev. Lett.* **71**, 2781–2784 (1993).

43. Y. He, M. Hashimoto, D. Song, S.-D. Chen, J. He, I. M. Vishik, B. Moritz, D.-H. Lee, N. Nagaosa, J. Zaanen, T. P. Devereaux, Y. Yoshida, H. Eisaki, D. H. Lu, Z.-X. Shen, Rapid change of superconductivity and electron-phonon coupling through critical doping in Bi-2212. *Science* **362**, 62–65 (2018).

44. J. Q. Lin, P. Villar Arribi, G. Fabbris, A. S. Botana, D. Meyers, H. Miao, Y. Shen, D. G. Mazzone, J. Feng, S. G. Chiuzbăian, A. Nag, A. C. Walters, M. García-Fernández, K.-J. Zhou, J. Pelliciari, I. Jarrige, J. W. Freeland, J. Zhang, J. F. Mitchell, V. Bisogni, X. Liu, M. R. Norman, M. P. M. Dean, Strong superexchange in a $d^{9-\delta}$ nickelate revealed by resonant inelastic x-ray scattering. *Phys. Rev. Lett.* **126**, 087001 (2021).

45. T. Valla, I. K. Drozdov G. D. Gu, Disappearance of superconductivity due to vanishing coupling in the overdoped $Bi_2Sr_2CaCu_2O_{8+\delta}$. *Nat. Commun.* **11**, 569 (2020).

46. S. P. Harvey, B. Y. Wang, J. Fowlie, M. Osada, K. Lee, Y. Lee, D. Li, H. Y. Hwang, Evidence for nodal superconductivity in infinite-layer nickelates. arXiv:2201.12971 [cond-mat.supr-con] (31 January 2022).

47. T. K. Kim, A. A. Kordyuk, S. V. Borisenko, A. Koitzsch, M. Knupfer, H. Berger, J. Fink, Doping dependence of the mass enhancement in $(Pb, Bi)_2Sr_2CaCu_2O_8$ at the antinodal point in the superconducting and normal states. *Phys. Rev. Lett.* **91**, 167002 (2003).

48. A. Kaminski, M. Randeria, J. C. Campuzano, M. R. Norman, H. Fretwell, J. Mesot, T. Sato, T. Takahashi, K. Kadowaki, Renormalization of spectral line shape and dispersion below T_C in $Bi_2Sr_2CaCu_2O_{8+\delta}$. *Phys. Rev. Lett.* **86**, 1070–1073 (2001).

49. B. J. Ramshaw, S. E. Sebastian, R. D. McDonald, J. Day, B. S. Tan, Z. Zhu, J. B. Betts, R. Liang, D. A. Bonn, W. N. Hardy, N. Harrison, Quasiparticle mass enhancement approaching optimal doping in a high-T_C superconductor. *Science* **348**, 317–320 (2015).

50. S. E. Sebastian, N. Harrison, G. G. Lonzarich, Towards resolution of the Fermi surface in underdoped high-T_C superconductors. *Rep. Prog. Phys.* **75,** 102501 (2012).

51. X. Chen, H. Zheng, D. P. Phelan, H. Zheng, S. H. Lapidus, M. J. Krogstad, R. Osborn, S. Rosenkranz, J. F. Mitchell, Competing charge/spin-stripe and correlated metal phases in trilayer nickelates $(Pr_{1-x}La_x)_4Ni_3O_8$. *Chem. Mater.* **10**, 4560–4567 (2022).
